# Automating Microinfarct Screening in Hematoxylin and Eosin‐stained Human Brain Tissues: A Machine Learning Approach

**DOI:** 10.1002/alz.095132

**Published:** 2025-01-09

**Authors:** Luca Cerny Oliveira, Joohi Chauhan, Zhengfeng Lai, Samson Cheung, Amparo Villablanca, Lee‐Way Jin, Charles S. DeCarli, Chen‐Nee Chuah, Brittany N Dugger

**Affiliations:** ^1^ University of California Davis, Davis, CA USA; ^2^ University of California Davis School of Medicine, Sacramento, CA USA; ^3^ University of California, Davis, Davis, CA USA; ^4^ University of California, Davis School of Medicine, Sacramento, CA USA; ^5^ University of California, Davis, Sacramento, CA USA

## Abstract

**Background:**

Microinfarcts, characteristic lesions of vascular dementia (VaD), are heterogenous and vary in appearance, which pose a considerable challenge for VaD grading as there is great interrater variability in microinfarct assessment. We propose a novel application of machine learning (ML) in the automated screening of microinfarcts, addressing a gap in the post‐mortem analysis of VaD in whole slide images (WSIs) from human brain.

**Method:**

Our study adapts a patch‐based pipeline with convolutional neural networks (CNNs) to automate microinfarct screening in WSIs. We compare performance and computational cost of different fields‐of‐view (FOV) for the patch‐based method. Additionally, we propose a postprocessing step and leverage multiple FOVs to mitigate false positives. This study is validated against 66 annotated WSIs (N = 40 infarcted, N = 26 un‐infarcted) from Frontal, Parietal, and Occipital white matter regions across 22 cases. Annotations are from a single trained expert, who delineated microinfarct regions and graded white matter rarefaction.

**Result:**

We report screening performance, i.e, the ability to distinguish infarct‐positive from infarct‐negative WSIs, and detection performance, i.e, the ability to localize the microinfarct regions within a WSI. Despite the inherent challenges of the inexact boundaries of microinfarcts, our models demonstrate notable efficacy in screening WSIs for infarcts, with ResNet‐18 achieving 100% accuracy at WSI level. However, the sensitivity in detecting infarct regions is below 50%, based on Intersection over Union thresholds above 20% overlap (a performance metric used to evaluate the accuracy of object detection models). Leveraging multiple FOV improves both detection and screening performances, reducing false positives.

**Conclusion:**

This work presents a proof‐of‐concept pipeline driven by machine learning to automate microinfarct screening & detection in WSIs. This workflow provides new avenues in the automated analysis of neuropathological images of microinfarcts, bringing potential advancement in ML‐based dementia and neuropathology research.

